# Differences in Radiation Exposure of CT-Guided Percutaneous Manual and Powered Drill Bone Biopsy

**DOI:** 10.1007/s00270-021-02851-z

**Published:** 2021-05-11

**Authors:** Sebastian Zensen, Sumitha Selvaretnam, Marcel Opitz, Denise Bos, Johannes Haubold, Jens Theysohn, Michael Forsting, Nika Guberina, Axel Wetter

**Affiliations:** 1 Department of Diagnostic and Interventional Radiology and Neuroradiology, University Hospital Essen, Hufelandstraße 55, 45147 Essen, Germany; 2 Department of Radiotherapy, University Hospital Essen, Hufelandstraße 55, 45147 Essen, Germany; 3 Department of Diagnostic and Interventional Radiology, Neuroradiology, Asklepios Klinikum Harburg, Eißendorfer Pferdeweg 52, 21075 Hamburg, Germany

**Keywords:** computed tomography, bone biopsy, radiation exposure, diagnostic reference level

## Abstract

**Purpose:**

Apart from the commonly applied manual needle biopsy, CT-guided percutaneous biopsies of bone lesions can be performed with battery-powered drill biopsy systems. Due to assumably different radiation doses and procedural durations, the aim of this study is to examine radiation exposure and establish local diagnostic reference levels (DRLs) of CT-guided bone biopsies of different anatomical regions.

**Methods:**

In this retrospective study, dose data of 187 patients who underwent CT-guided bone biopsy with a manual or powered drill biopsy system performed at one of three different multi-slice CT were analyzed. Between January 2012 and November 2019, a total of 27 femur (**A**), 74 ilium (**B**), 27 sacrum (**C**), 28 thoracic vertebrae (**D**) and 31 lumbar vertebrae (**E**) biopsies were included. Radiation exposure was reported for volume-weighted CT dose index (CTDI_vol_) and dose–length product (DLP).

**Results:**

CTDI_vol_ and DLP of manual versus powered drill biopsy were (median, IQR): **A**: 56.9(41.4–128.5)/66.7(37.6–76.2)mGy, 410(203–683)/303(128–403)mGy·cm, **B**: 83.5(62.1–128.5)/59.4(46.2–79.8)mGy, 489(322–472)/400(329–695)mGy·cm, **C**: 97.5(71.6–149.2)/63.1(49.1–83.7)mGy, 627(496–740)/404(316–515)mGy·cm, **D**: 67.0(40.3–86.6)/39.7(29.9–89.0)mGy, 392(267–596)/207(166–402)mGy·cm and **E**: 100.1(66.5–162.6)/62.5(48.0–90.0)mGy, 521(385–619)/315(240–452)mGy·cm. Radiation exposure with powered drill was significantly lower for ilium and sacrum, while procedural duration was not increased for any anatomical location. Local DRLs could be depicted as follows (CTDI_vol_/DLP): **A**: 91 mGy/522 mGy·cm, **B**: 90 mGy/530 mGy·cm, **C**: 116 mGy/740 mGy·cm, **D**: 87 mGy/578 mGy·cm and **E**: 115 mGy/546 mGy·cm. The diagnostic yield was 82.4% for manual and 89.4% for powered drill biopsies.

**Conclusion:**

Use of powered drill bone biopsy systems for CT-guided percutaneous bone biopsies can significantly reduce the radiation burden compared to manual biopsy for specific anatomical locations such as ilium and sacrum and does not increase radiation dose or procedural duration for any of the investigated locations.

**Level of Evidence:**

Level 3.

## Introduction

CT-guided percutaneous bone biopsies play a key role for the diagnostic work-up of skeletal lesions such as inflammatory and malignant processes. Compared with standard open biopsy, CT-guided approaches are less invasive and provide a sufficient specimen yield [1–3]. Furthermore, CT-guided biopsies have a lower complication rate and are generally well tolerated [4, 5]. These procedures can be performed either with a manual approach by placing a bone needle within the skeletal lesion, possibly achieved by hammering technique to perforate the cortical bone, or with a powered drill bone biopsy system such as the commercially available Arrow® OnControl® powered bone access system. For this battery-powered drill, decreased procedural duration, improved user-friendliness and lower pain perception were reported [6, 7]. Furthermore, the powered drill approach provides a higher diagnostic yield for sclerotic lesions [4]. Alongside these benefits of manual and powered drill CT-guided bone biopsies, CT entails a radiation burden and is a high-dose imaging technique, which causes the major part of collective effective dose of all medical imaging [8, 9]. While some recent studies reported radiation doses of CT-guided bone biopsies [4, 10–12], further detailed dose assessment and comparison between different anatomical regions are needed to optimize radiation protection. Additionally, specific reports of diagnostic reference levels (DRL) are rare [13]. For various indications, DRLs were established to limit radiation exposure of radiological imaging modalities [14]. To compare and evaluate local radiation exposure distributions and optimize radiation protection, 75^th^ percentiles of dose metric distributions are often used as DRL [15].

The aim of this study was to evaluate the radiation exposure and procedural duration of CT-guided percutaneous manual and powered drill bone biopsies and to establish local DRLs.

## Materials and Methods

### Patient Cohort

Between January 2012 and November 2019, dose data of all CT-guided percutaneous bone biopsies at our center were included, which provided full information for dose metrics, precisely reported anatomical location as well as technical and procedural duration information. Patients were identified using the radiological information system (RIS). Following anatomical locations were included: femur, ilium, sacrum, thoracic and lumbar vertebrae. Clinical information was extracted from the report archived in the RIS. Ethical approval for this retrospective single-center study was granted by the institutional review board and the requirement to obtain informed consent was waived (19–8579-BO).

### CT Scanners and Biopsy Equipment

**Fig. 1:**
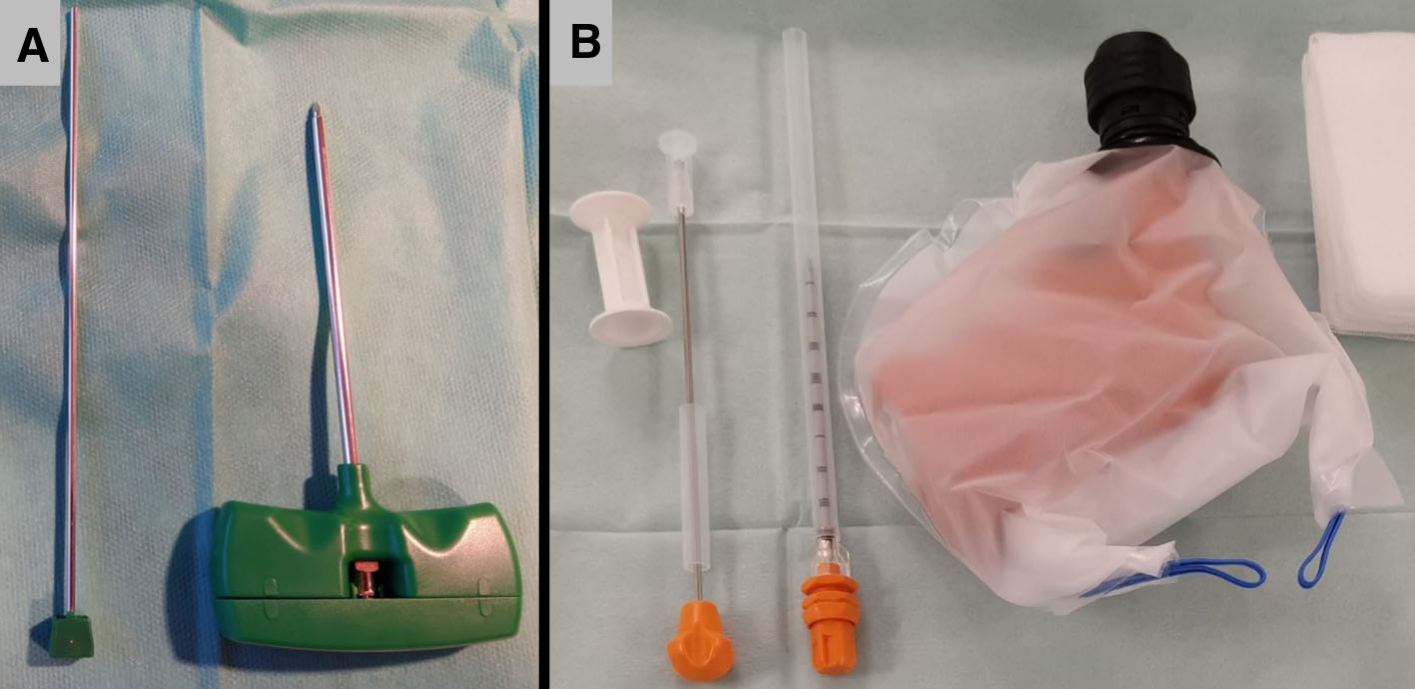
Manual and powered drill bone biopsy systems. Shown in **A** is a typical commercially available manual bone biopsy kit consisting of the biopsy needle (right) and an obturator (left) for pushing the specimen out of the needle. Shown in **B** is the reusable, non-sterile battery-powered drill (Arrow OnControl, Teleflex, Wayne, USA) (right), which was wrapped in a sterile bag prior to biopsy, and an associated disposable 11-gauge 4 inch (102 mm) biopsy needle (center) and an obturator (left) for pushing the specimen out of the needle

All interventions were performed by experienced interventional radiologists at one of three commercially available, modern multi-slice CT scanners: single-source 128-slice SOMATOM Definition AS + , dual-source 128-slice SOMATOM Definition Flash and dual-source 192-slice SOMATOM Force (all: Siemens Healthineers, Erlangen, Germany). At dual-source CT scanners, only one tube was used. For all scans, the tube voltage was 120 kV and the rotation time 0.5 s. Further technical settings according to CT scanner are shown in Table 1. For manual bone biopsy, commercially available 10-, 11- or 13-gauge bone biopsy needles such as the Ostycut® bone biopsy needle (Bard, Covington, USA) and other bone biopsy sets (Stryker, Kalamazoo, USA) were used. For battery-powered drill bone biopsy, the Arrow® On Control® powered bone access system with attachable 11-gauge biopsy needle (Teleflex, Wayne, USA) was applied, which is a handheld powered drill with electric drive and manual guidance of the drilling channel (Fig. 1).

**Table 1:** Technical parameters of CT-guided percutaneous bone biopsy at three different multi-slice Siemens CT scanners

CT scanner*	Femur			Ilium			Sacrum			Thoracic vertebrae			Lumbar vertebrae		
	AS +	Flash	Force	AS +	Flash	Force	AS +	Flash	Force	AS +	Flash	Force	AS +	Flash	Force
*n*	14	11	2	16	45	13	4	17	6	11	15	2	8	18	5
Tube current–time product (mAs)	50	50	150	50	50	150	50	50	150	50	50	165	50	50	150
Slice thickness (mm)	1.2	1.2	5.0	1.2	1.2	5.0	1.2	1.2	5.0	1.2	1.2	1.0/5.0	1.2	1.2	2.5

^*^AS + : SOMATOM Definition AS + ; Flash: SOMATOM Definition Flash; Force: SOMATOM Force (all: Siemens Healthineers, Erlangen, Germany)

### Definitions

To determine the diagnostic yield of the CT-guided bone biopsies, the pathology reports were checked to see if a diagnosis could be made from the specimen. A biopsy was considered diagnostic if the specimen was eligible for histological evaluation.

To investigate the dose difference between bone biopsies of osteolytic and osteoblastic lesions, the average density of the lesion was determined. An average density below 250 HU was considered osteolytic, above that osteoblastic.

Bone biopsies were divided into superficial and deep biopsies according to the depth of the lesion, which was measured from the skin puncture site to the site of the tip of the biopsy needle or drill bit within the bone lesion. A depth up to 70 mm was considered superficial, above that depth was considered deep.

The assessed procedural duration refers to the period between the start of the intra-procedural, i.e., biopsy-guiding sequence with acquisition of the first scan after puncture to its end with successful placement of the biopsy needle in the lesion but before acquisition of the post-biopsy scan for documentation and recording of possible complications.

### Bone Biopsy Procedure

First, a prebioptic scan was obtained for biopsy planning. Subsequently, the area of the planned puncture site was locally anesthetized. The manual system contains a disposable biopsy cannula with internal stylet. Intraosseously, the inner stylet was removed and the biopsy needle advanced through the lesion to the desired depth using rotary motion or hammering technique using a mallet. Subsequently, the system was completely removed and the specimen was carefully extruded from the needle using an obturator. The powered drill system includes a handheld reusable electric drill with a sealed lithium-ion battery to which a disposable bone biopsy needle is attached. The drill does not have a hammer function and the attachable 11-gauge biopsy needle is available in a length of 4 or six inches (102 mm or 152 mm) and is coaxial in design with an outer cannula and an inner stylet with a beveled tip [12]. The non-sterile drill was wrapped in a sterile bag prior to biopsy and connected to the biopsy needle via a connector. Once the needle tip was placed immediately in front of the bone lesion, the drill was removed from the connector and the inner stylet was removed from the biopsy needle. The drill was then reconnected and the biopsy needle was used to drill through the bone lesion to the desired depth. The system was then completely removed and the specimen in the biopsy needle was carefully pushed out using the stylet. In both approaches, intermittent biopsy-guiding CT scans were taken for positional control. As soon as the biopsy needle could be delineated intralesionally, the internal stylet was removed and the tissue sample was collected. The biopsy system was then removed and a post-bioptic scan was performed for documentation and to exclude complications.

### Dose Assessment

For dose assessment, examination data and dose measurements were extracted from the Digital Imaging and Communications in Medicine (DICOM) header and from the Radiation Dose Structured Report stored in the Picture Archiving and Communication System (PACS). Dose assessments referred to the 32 cm diameter standard polymethyl methacrylate (PMMA) CT dosimetry phantom. Assessed radiation exposure indices were the volume-weighted CT dose index (CTDI_vol_) and dose–length product (DLP). Although they do not directly represent the dose to an individual patient, CTDI_vol_ and DLP quantify the radiation dose output of a CT scanner and may help to ensure lower radiation exposures. DRLs were set at the 75^th^ percentile of dose distribution. Both the manual and powered drill bone biopsies were performed under CT guidance in step-and-shoot technique. For both approaches, multiple CT scans were needed to monitor the location of the biopsy needle, respectively, the drill tip during the biopsy-guiding scans. All DLP values of the biopsy-guiding scans were added to a total DLP, so that all scans necessary for the biopsy and possibly acquired CT spirals were included in the total DLP. To emphasize the differences of radiation dose contributed to the application of manual versus powered drill bone biopsy system, radiation doses of biopsy-guiding scans were analyzed additionally with exclusion of pre- and post-bioptic scans. Dose assessment was also performed for different subgroups in relation to characteristics of the bone lesions, that is, in terms of density, depth, anatomical location, suspected etiology, and technical parameters such as needle diameter and protocols on CT scanners.

### Statistics and Data Analysis

Descriptive statistics were performed using GraphPad Prism 5.01 (GraphPad Software, San Diego, USA). To determine normal distribution Kolmogorov–Smirnov, Shapiro–Wilk and D’Agostino–Pearson test were applied. Normally distributed data are reported as mean ± standard deviation (SD), non-normally distributed data as median and interquartile range (IQR). Mann–Whitney U test was applied to compare radiation indices between manual and powered drill approaches. Kruskal–Wallis test with Dunn–Bonferroni post hoc test was performed to compare procedural durations and DLP values of manual biopsies with different needle diameters. A p value lower than 0.05 was considered statistically significant.

## Results

### Patient Cohort

In our retrospective study, 187 patients who underwent a CT-guided percutaneous bone biopsy between January 2012 and November 2019 could be included for evaluation. Median age was 57.4 years (IQR 42.9–67.6, total range 7.9–86.5 years). Median BMI of patients with manual biopsy was 25.0 kg/m^2^ (IQR 22.8–28.0) and of patients with powered drill biopsy 24.2 kg/m^2^ (IQR 22.1–27.1). Statistical analysis showed that there was no significant difference in BMI (*p* = 0.3065). Included datasets comprised a total (manual/powered drill) of 27 (13/14) femur (**A**), 74 (27/47) ilium (**B**), 27 (11/16) sacrum (**C**), 28 (13/15) thoracic vertebrae (**D**) and 31 (10/21) lumbar vertebrae (**E**) biopsies. Regarding diagnostic yield, 82.4% (61 out of 74) manual and 89.4% (101 out of 113) powered drill bone biopsies provided sufficient specimen yield and quality.

### Radiation Exposure of Manual and Powered Drill Bone Biopsy in Relation to Anatomical Localization

**Fig. 2:**
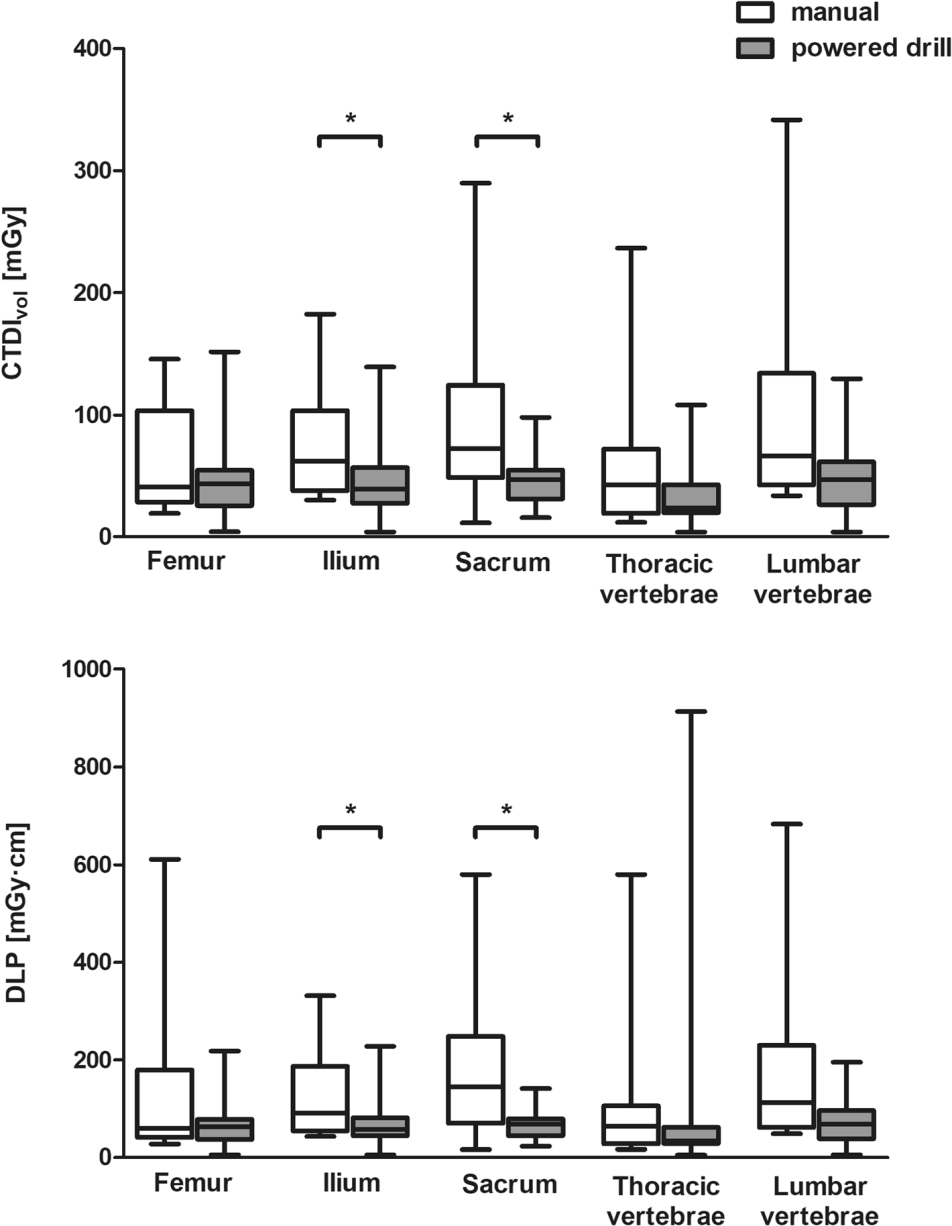
Distribution of volume-weighted CT dose index (CTDI_vol_) and dose–length product (DLP) of CT-guided percutaneous bone biopsy with manual and battery-powered drill system for different anatomical locations. Highlighting radiation exposure indices of biopsy-guiding scans with exclusion of radiation exposure of topogram, pre- and post-bioptic scans to emphasize radiation exposure differences between manual and battery-powered drill bone biopsy approaches. Whiskers represent min to max. Asterisks indicate significant difference (*p* < 0.05)

28% (53 out of 187) of all procedures were performed at SOMATOM AS + , 57% (106 out of 187) at SOMATOM Definition Flash and 15% (28 out of 187) at SOMATOM Force. Major part of radiation indices values, all median CTDI_vol_ values except for ilium and all median DLP values for the whole procedure including pre-bioptic scans for planning, the biopsy-guiding scans and the post-bioptic scans for documentation and exclusion of immediate complications of the biopsy were lower with powered drill biopsy (Tables 2 and 3). Similar to the radiation dose distribution of the whole procedure, the major part of median CTDI_vol_ and DLP values as well as IQRs of the biopsy-guiding scans were lower with the powered-drill approach (Fig. 2). Statistical analysis revealed significantly lower CTDI_vol_ for biopsy-guiding scans of powered-drill biopsies of ilium (*p* < 0.0001) and sacrum (*p* = 0.0232). Likewise, radiation exposure in terms of DLP for biopsy-guiding sequences was significantly lower for both anatomical regions: ilium (*p* = 0.0008), sacrum (*p* = 0.0178). No statistical significant difference was found for other biopsy regions.

**Table 2:** Volume-weighted CT dose index (CTDI_vol_) of CT-guided percutaneous bone biopsy with manual and battery-powered drill system for different anatomical regions

Anatomical location		n	CTDI_vol_ [mGy]			
			25th percentile	Median	75th percentile	Mean ± SD
Femur	Total	27	42.4	65.4	90.9	71.0 ± 43.1
	Manual	13	41.4	56.9	128.5	80.9 ± 47.7
	Powered drill	14	37.6	66.7	76.2	61.8 ± 37.8
Ilium	Total	74	50.4	71.5	90.3	80.0 ± 41.7
	Manual	27	62.1	83.5	128.5	101.3 ± 49.2
	Powered drill	47	46.2	59.4	79.8	67.8 ± 31.1
Sacrum	Total	27	58.4	71.6	115.5	92.3 ± 59.2
	Manual	11	71.6	97.5	149.2	124.9 ± 76.6
	Powered drill	16	49.1	63.1	83.7	69.9 ± 29.0
Thoracic vertebrae	Total	28	34.1	48.3	86.6	64.6 ± 49.6
	Manual	13	40.3	67.0	86.6	75.7 ± 61.4
	Powered drill	15	29.9	39.7	89.0	54.9 ± 35.9
Lumbar vertebrae	Total	31	52.0	70.9	115.0	91.1 ± 68.2
	Manual	10	66.5	100.1	162.6	133.0 ± 97.7
	Powered drill	21	48.0	62.5	90.0	71.2 ± 37.3

**Table 3:** Dose–length product (DLP) of CT-guided percutaneous bone biopsy with manual and battery-powered drill system for different anatomical regions

Anatomical location		n	DLP [mGy·cm]			
			25th percentile	Median	75th percentile	Mean ± SD
Femur	Total	27	179.3	332.0	522.0	366.3 ± 212.6
	Manual	13	203.1	410.0	682.8	442.5 ± 250.3
	Powered drill	14	127.7	302.8	403.1	295.5 ± 146.3
Ilium	Total	74	327.7	419.8	529.8	458.9 ± 211.1
	Manual	27	322.0	489.0	471.8	524.7 ± 255.0
	Powered drill	47	328.5	399.7	695.0	421.0 ± 173.2
Sacrum	Total	27	341.0	492.6	740.0	537.0 ± 248.2
	Manual	11	496.0	626.6	740.0	659.3 ± 287.6
	Powered drill	16	316.1	403.7	515.3	453.0 ± 182.0
Thoracic vertebrae	Total	28	193.5	315.7	577.7	418.3 ± 331.6
	Manual	13	267.0	392.0	596.0	473.3 ± 316.3
	Powered drill	15	165.8	206.9	402.1	370.6 ± 347.9
Lumbar vertebrae	Total	31	263.4	402.0	545.8	437.2 ± 259.2
	Manual	10	384.8	520.5	619.0	559.0 ± 279.8
	Powered drill	21	240.4	314.5	452.1	379.2 ± 233.7

### Comparison Between Different Protocols on the CT Scanners

Because of the higher tube current product on the SOMATOM Force, a subgroup analysis was performed with all manual versus powered drill biopsies on SOMATOM AS + and Flash: Median DLP of biopsy-guiding sequence was similar for manual biopsies with 60.7 mGy·cm (IQR 44.0–85.5) and for powered drill biopsies with 59.6 mGy·cm (IQR 39.3–78.9) and showed no significant difference (*p* = 0.04383). In contrast, a median DLP of 218 mGy·cm (IQR 172.8–311.0) was obtained for all manual biopsies on the SOMATOM Force. Manual biopsies on the SOMATOM Force required significantly higher DLP values compared to both SOMATOM AS + and Flash (*p* < 0.0001).

### Influence of Bone Lesion Characteristics on Radiation Exposure

Regarding the density of the bone lesion, no significant difference of median DLP values was detected in both manual osteolytic (101.0 mGy·cm; IQR 52.0–207.3) and osteoblastic (86.0 mGy·cm; IQR 64.0–124.0) biopsies (*p* = 0.6526). Similarly, the doses of powered drill biopsies did not differ significantly at a median DLP of 62.0 mGy·cm (IQR 44.6–81.0) for osteolytic and 66.0 mGy·cm (IQR 43.4–78.9) for osteoblastic lesions (*p* = 0.8592). In contrast, the choice of biopsy system for both osteolytic (*p* = 0.0007) and osteoblastic (*p* = 0.0105) bone lesions showed significantly lower DLP values for the powered drill biopsies. In terms of depth of bone biopsy, the radiation exposure of the biopsy-guiding scans of the manual biopsies was significantly lower for the superficial biopsies with a median DLP of 53.5 mGy·cm (IQR 45.3–64.0) than for the deep biopsies with 88.0 mGy·cm (IQR 81.0–98.0) (*p* < 0.0001). Similarly, the median DLP values of the superficial powered drill biopsies (44.6 mGy·cm, IQR 29.0–67.6) were significantly lower than those of the deep biopsies (73.5 mGy·cm, IQR 51.1–91.6) (*p* < 0.0001). In addition, dose values in terms of DLP were significantly lower with powered drill compared with manual biopsy for both superficial (*p* = 0.0002) and deep (*p* = 0.0441) biopsies. The distribution of the suspected etiology of the bone lesions could be determined as follows (manual/powered drill): 21.6%/15.9% primary bone tumor, 75.7%/79.7% bone metastasis, 2.7%/4.4% inflammatory process. There was no difference in both manual and powered drill biopsies with respect to DLP in the biopsy-guiding scans (*p* = 0.903, *p* = 0.555), excluding the inflammatory bone lesions due to too small sample size. When comparing between biopsy systems, there were significantly lower DLP values when metastases were biopsied with the powered drill (median 59.8 mGy·cm, IQR 42.4–78.7) compared to manual biopsy (median 85.0 mGy·cm, IQR 50.0–202.0) (*p* = 0.0002). In contrast, radiation exposure for biopsy of primary bone tumors did not differ significantly between the manual (median 95.5 mGy·cm, IQR 48.8–182.8) and powered drill (median 72.9 mGy·cm, IQR 37.8–98.6) system (*p* = 0.1379). All included biopsies were performed by one of two interventional radiologists with several years of experience with both biopsy systems. In terms of an interoperator variability on radiation exposure, no trend was observed in terms of diagnostic yield or dose differences. Regarding the different needle diameters used in the manual bone biopsies of 10G, 11G and 13G, Kruskal–Wallis test with Dunn–Bonferroni post hoc test showed no significant difference in the DLP values of the biopsy-guiding scans (*p* = 0.5599).

### Establishment of Local Diagnostic Reference Levels

Local DRLs at our institution for CT-guided bone biopsies could be depicted as follows (CTDI_vol_/DLP): **A**: 91 mGy/522 mGy·cm, **B**: 90 mGy/530 mGy·cm, **C**: 116 mGy/740 mGy·cm, **D**: 87 mGy/578 mGy·cm and **E**: 115 mGy/546 mGy·cm.

### Procedural Duration of CT-guided Percutaneous Bone Biopsies

Lowest median procedural duration of the CT-guided bone biopsy with exclusion of pre- and post-bioptic scans was depicted for ilium with 21.0 (IQR 16.6–27.7) minutes, highest for lumbar vertebrae with 23.3 (IQR 17.5–32.1) minutes (Table 4). Comparing manual and powered drill approaches, median procedural durations were between 2.2 (**A**) and 6.6 min (**E**) less for powered drill approaches. Nonetheless, Kruskal–Wallis test revealed no significant difference between subgroups (*p* = 0.512). No significant difference in duration was also found with respect to the density of the bone lesion (*p* = 0.1477).

**Table 4:** Duration of CT-guided percutaneous bone biopsy with manual versus battery-powered drill system at different anatomical regions

Region		n	Duration [min]	
			Median	IQR
Femur	Total	27	22.7	16.4–27.9
	Manual	13	24.7	18.6–40.5
	Powered drill	14	22.5	16.3–26.3
Ilium	Total	74	21.0	16.6–27.7
	Manual	27	23.5	16.8–31.3
	Powered drill	47	19.2	15.0–26.8
Sacrum	Total	27	21.9	15.7–26.4
	Manual	11	25.1	15.7–29.2
	Powered drill	16	20.6	15.9–24.2
Thoracic vertebrae	Total	28	22.0	11.5–31.7
	Manual	13	23.4	14.4–31.7
	Powered drill	15	18.7	9.7–32.7
Lumbar vertebrae	Total	31	23.3	17.5–32.1
	Manual	10	28.7	16.9–39.7
	Powered drill	21	22.1	18.1–30.3

## Discussion

In this study, the comparison of manual and powered drill CT-guided percutaneous bone biopsies revealed significantly lower radiation exposure for biopsy-guiding scans of ilium and sacrum with a powered drill biopsy system, and radiation exposure indices were also slightly lower for the other evaluated anatomical locations. Furthermore, our study demonstrated that for both osteolytic and osteoblastic bone lesions, as well as for superficial and deep biopsies, radiation exposure was lower with the powered drill system. Hence, further dose reduction in CT-guided bone biopsies is achievable by using powered drill biopsy systems. Furthermore, a slight but not significant decrease in procedural duration could be depicted for the powered drill approaches.

Bone biopsies play a key role for several diseases causing skeletal lesions such as infectious or malignant processes [10]. CT guidance is used for biopsies of many anatomical locations within the body as it improves identification of a pathology, enables planning of access route through the body and reduces costs and interventional risks compared with open biopsy [16]. Battery-powered drill bone biopsy systems such as the commercially available Arrow® OnControl® rotatory drill pose an alternative approach to place a bioptic needle in the desired depth within a skeletal lesion opposed to the manual approach with inserting the needle by manual pressure and hammering technique [7, 17]. However, the main objective of a CT-guided percutaneous biopsy, whether using a manual or powered drill biopsy system, is to provide a sufficient amount of biopsy material, and thus the diagnostic yield should be equivalent for both approaches as a matter of priority. In this coherence, several studies reported a sufficient diagnostic yield of both manual and powered drill CT-guided bone biopsies [4, 10]. Our results showed that a high diagnostic yield, comparable to other studies, was given for both approaches and was slightly higher for the powered drill biopsies. Nevertheless, differences in radiation burden and procedural duration might optimize patient care and minimize radiation exposure [10]. Therefore, radiation protection aspects of CT-guided bone biopsies are worth to consider. Several studies reported dose assessments: For example, Yang et al. reported radiation exposures with a median DLP of 733 mGy·cm (IQR 462–1086 mGy·cm) [11]. Our results, like the study by Lee et al. comparing manual and powered drilling systems (mean ± SD CTDI_vol_: manual 270 ± 48 mGy, powered drill 164 ± 35 mGy), demonstrated that the radiation exposure was significantly lower with the powered drill approach [12]. In contrast, it also has been reported that radiation exposure was slightly higher with the power drill (DLP 1203 mGy·cm) than with the manual biopsy system (DLP 971 mGy·cm) [4].

Various factors such as the anatomical location and etiology of the bone lesion, as well as the choice of biopsy system, influence the diagnostic yield of CT-guided bone biopsies [18–21]. In this context, different features also influence radiation exposure of the biopsy procedure. For example, densely and sclerotic lesions are more difficult to be attained both with manual needle and powered drill [18, 19]. Therefore, more biopsy-guiding CT scans are likely to be required in such cases, increasing the radiation exposure and also procedural duration [10]. Kihira et al. reported radiation exposure of bone biopsies differentiated by density to be higher for manual than for powered drill biopsies with mean DLP values between 752 and 1317 mGy·cm [10]. In our study, the results demonstrated that the density of a bone lesion, i.e., osteolytic or osteoblastic, had no significant effect on radiation exposure, but the use of the biopsy system did. With regard to an interoperator variability, our study showed no significant differences in the radiation exposures of the bone biopsies. In addition to diagnostic yield and radiation exposure, characteristics of the bone lesion and the biopsy system are also thought to influence procedural duration. Comparable to the results of Cohen et al., our study demonstrated that procedural duration of all evaluated anatomical locations was slightly lower with the powered drill biopsy system, although this time saving was not significant [4]. Although the powered drill approach is reported to offer shorter scanning time for biopsies of densely sclerotic lesions in addition to less specimen artifacts [10, 18], no significant difference in procedural duration between biopsies of osteolytic and osteoblastic lesions was observed in our study. Aside from considerations related to radiation exposure and duration, many factors influence the choice of a bone biopsy system such as availability, costs and operator preference [10]. Therefore, not only do local preferences differ with respect to biopsy systems and procedures, but also the radiation exposures of CT examinations can vary significantly by institution [20, 21]. Helpful benchmarks for dose monitoring are DRLs which indicate typical ionizing radiation exposure values in a country, region or an institute [22]. Although the establishment of DRLs for CT interventions is more difficult compared to diagnostic examinations due to a wide variation in location and technique, the establishment of DRLs might play a crucial role for dose optimization in interventional radiology. However, not only European and national DRLs for CT-guided bone biopsies are lacking, but also reports of locally established DRLs are rare. Therefore, our local DRLs for CT-guided bone biopsies of the most common anatomical locations might be an another step toward the establishment of national or European DRLs.

Limitations of our study are the retrospective design and that there were no equivalent numbers of manual and powered drill biopsies and partly different protocols on the CT scanners. With regard to the CT scanners used, comparison of manual biopsies on SOMATOM Force versus biopsies on SOMATOM AS + and Flash with same settings showed that DLP values were significantly higher on the SOMATOM Force. Accordingly, comparable settings on all scanners and same number of cases would be a significant optimization factor. Strengths of our study include the detailed dose assessment, which enables detailed evaluation on radiation dose of manual versus powered drill biopsy approaches. Furthermore, several anatomical locations and procedural durations were evaluated.

## Conclusion

In conclusion, our study demonstrated that the use of a powered drill bone biopsy system for CT-guided percutaneous bone biopsy can reduce the radiation exposure significantly for specific anatomical locations. For both osteolytic and osteoblastic bone lesions, as well as for superficial and deep biopsies, radiation exposure was lower with the powered drill system. DRLs for CT-guided bone biopsies are needed to optimize radiation protection, and our locally determined DRLs may help as benchmarks.
